# From super-wicked problems to more-than-human justice: new bioethical frameworks for antimicrobial resistance and climate emergency

**DOI:** 10.1007/s40592-024-00197-z

**Published:** 2024-07-11

**Authors:** Tiia Sudenkaarne, Andrea Butcher

**Affiliations:** Department of Sociology, University of Helsinki, Helsinki, Finland

**Keywords:** More-than-human, Social study of microbes, Super-wicked problems, One health, Queer, Feminist, Posthuman

## Abstract

In this article, building on our multidisciplinary expertise on philosophy, anthropology, and social study of microbes, we discuss and analyze new approaches to justice that have emerged in thinking with more-than-human contexts: microbes, animals, environments and ecosystems. We situate our analysis in theory of and practical engagements with antimicrobial resistance and climate emergency that both can be considered super-wicked problems. In offering solutions to such problems, we discuss a more-than-human justice orientation, seeking to displace human exceptionalism while still engaging with human social justice issues. We offer anthropological narratives to highlight how more-than-human actors already play an important role in environmental and climate politics. These narratives further justify the need for new ethical frameworks, out of which we, for further development outside the scope of this article, suggest a queer feminist posthumanist one.

## Introduction: conceptual biodiversity of justice

Our inquiry was initiated by the observation that in many recent networking events with social scientific scholars working on antimicrobial resistance (AMR), conversations seemed to include parallels between AMR and climate emergency (CE). There is a growing understanding of AMR and CE as somehow equally bioethically demanding: both are highly complex with various more-than-human stakeholders yet often dealt with by human exceptionalism, yet both also grossly compound the reproduction of binaries between existing social justice and environmental justice issues, such as the division between the global North and South reproducing racial capitalism (e.g. Sariola et al. 2022; see also Gonzalez 2021) By human exceptionalism, we refer to the anthropocentric notion of considering human ways of being, doing and making sense of the world more valuable than others (e.g. Celermajer et al. 2021), albeit as humans we acknowledge our limitations in dismantling this exceptionalism. In this article, we interrogate the bioethical parallels between AMR and CE by framing them as super-wicked problems and asking, how could justice as a bioethical principle guide to alleviate them. There is both practical and institutional support for a principlist approach in dealing with CE ethical issues: in its 2017 declaration, UNESCO builds on the notion that “climate change not only threatens our ecosystems, it undermines the foundation of our fundamental rights, deepens inequalities and creates new forms of injustice”. This global initiative is to us an excellent nudge to reconfigure justice, one of the four prominent bioethical principles (Beauchamp and Childress 2019). However, it is crucial to note that for an ethically sustainable definition and application of justice as a principle, new frameworks are needed to address AMR and CE without human exceptionalism while also keeping social justice in the same framework. By framework, we mean a cohesion-seeking theoretical approach and practical applications with ontological, political and ethical aims (cf. queer bioethics as a moral theory) (e.g. Sudenkaarne 2021; 2022). By orientation, we mean an approach that looks at a specific elemental concept within that framework We suggest towards a queer feminist posthuman framework as one of the new ethical frameworks needed to reframe justice. However, as it is not possible to deliver a full framework in an article, this work is taken on more thoroughly elsewhere (cf. Author).

Another important grounding for our approach is the growing, shared, cross-disciplinary interest in the concept of justice (cf. Chao et al. 2022). We will begin by briefly addressing this conceptual biodiversity to offer our distinct approach. By framing AMR and CE as super-wicked problems, we seek to demonstrate the limits of human exceptionalist frameworks for and definitions of justice, offering anthropological narratives throughout this treatment to make our case. We conclude that there is an ontological relationality between AMR and CE: both are connected through and driven by human action albeit at the brink of breaking from all human control. Thus, justice considerations must decentralize the human, and new ethical frameworks are needed to centralize more-than-human.

We argue that current ethical understanding driving policies of management and prevention of AMR and CE are building on a human exceptionalist premise that is not only harmful but also inaccurate in reflecting the role of more-than-human in both. The World Health Organization (WHO) has declared them amongst “the top ten global public health threats facing humanity” (2021). In common understanding, AMR occurs when bacteria, viruses, fungi and parasites change over time and no longer respond to medicines, making infections harder to treat and increasing the risk of disease spread, severe illness and death. However, this dominant understanding of AMR only covers one aspect of microbial resistance: the safeguarding of antimicrobial therapies for continued protection of *human* health. This definition does not offer an appreciation of more-than-human (animals, microbes, ecosystems, environments) *that can also* address human health without prioritizing it (Cañada et al. 2022).

Based on experiencing the 2023 summer temperatures in Europe alone, growing concerns over accelerating extreme weather unpredictability have shifted the discourse from climate change to climate *emergency* (McHugh et al. 2021). From draught causing erosion and forest fires to excess rainfall, heat waves, hurricanes, and floods there is a vicious cycle between climate change, reliance on human structures such as fossil capitalism (Malm 2016), human health and wellbeing through effects on weather, ecosystems, and human systems (Nuffield Council on Bioethics 2023). As the 2021 report of the Lancet Countdown on health and climate change (see also Nuffield Council on Bioethics 2023, 4) states:

These effects increase exposure to extreme events, change the environmental suitability for infectious disease transmission, alter population movements and undermine people’s livelihoods and mental health. The resulting strain on health and social system disproportionately affects the most disadvantaged in society.

Similar urgency is expressed in global AMR governance, referring to the increasing abundance and proliferation of resistance genes across the planet as the “silent pandemic” (Gerhard 2021; Akram et al. 2023; World Bank 2019); or an “antibiotic apocalypse” (McKie 2017a, b; Nerlich and James 2009), undermining sustainable development gains, harming economies, and costing millions of lives (World Bank 2019).

Echoing the notion of intertwined health, The UN Secretary-General, António Guterres (2020) has stated that is “the time to save the sick and rescue the planet.” Despite the appeal of such a slogan, similarly to framing AMR as environmental problem because its environmental effects threaten human health and wellbeing, this approach is insufficient in grounding a more-than-human justice orientation as more-than-human actors such as environments are considered instrumental to human flourishing rather than seeking planetary flourishing. Thus we suggest an approach building on “radical reorientation” of human exceptionalism (Cañada et al. 2022, 2).

In this reorientation, we join those who promote justice as a key concept, but it needs to be acknowledged there are different approaches. Sharing some of our premises, like building on relationality and refusing human exceptionalism, multispecies justice is a concept currently receiving much attention. Building on the concept originated by Haraway (2008), to Chao and Kirksey (2022, 99), it is required to stay anchored in the open, alive generative possibilities of each more-than-human encounter; it is an approach that demands “us” to decide which dreams are worth dreaming—and by extension, which injustices are intolerable. In addition to justice-orientation aligned with original frameworks, there are also approaches that operate from a legal rights framework, seeking the recognition of new more-than-human subjects with rights such as the rights of the Amazonas ecosystem (Lyons 2022). However, as Lyons (2022, 53) observes, more capacious rights frameworks are not immune to injustice when they deny the voices and realities of communities most implicated in the impacts of their judicial decisions.

In a multispecies framework, there seems to be a clash between animal rights thinking on the one hand, and an environmental justice approach on the other, as there is an ongoing tension between animal rights positions’ focus on the irreducible good of the individual, and environmental holists’ focus on the functioning of systems (Celermajer et al. 2021, 121). Such theoretical challenges motivate considerations of multispecies justice (Celermajer et al. 2021, 121–122; Lyons 2022; Wienhues 2020; Boetzkes and Robert 2000). Further, the intellectual and activist impetus under the animal rights umbrella, arguing from utilitarian or deontological ethics, rests heavily on the sentience argument (Celermajer et al. 2021, 122). Briefly, it builds on a hierarchy for life forms in which those most sentient are at the top and those considered the least sentient and (at least to human cognition) most distant in their sense-making, at the bottom. In this line of thinking, justice requires teleological considerations about what is good for an individual of a certain species, such as ending use of animals in agriculture (Animal Justice Project 2023), and how living things can be granted rights based on their intrinsic value (Celermajer et al. 2021; Boetzkes and Robert 2000).

The global AMR discourse and rhetoric similarly introduces value hierarchies as the multispecies justice model discussed above, privileging the protection of human and public health, with animals and environments valued only to the extent that they support the safety and flourishing of human bodies and wellbeing. Whilst the kinds of multispecies frameworks discussed above would seemingly apply well to AMR, given the shared microbiota between humans and nonhumans (and of course microbiota as particular kinds of nonhumans themselves), theorization of social justice due to the impacts of AMR remain limited to protection of vulnerable *human* populations, for whom interventions such as limiting access to antibiotics risk increasing their vulnerability in terms of loss of healthcare access or livelihoods (in the case of food production) (Broom et al. 2021; Littman 2014; Varadan et al. 2023). One can speculate that this is partly due to the dominant framing of the AMR problem as one of therapeutic failure due to purported ill-informed individual decision-making (e.g. patient, clinician, veterinarian or food producer) when resorting to antibiotic therapies, a framing which these authors challenge, whereas the climate emergency is framed as one of collective pollution. In one exception, Kirchhelle’s 2023 thought piece calls for an examination of the ecosocial injustices that result from unequal antimicrobial exposure resulting from industrial, pharmaceutical and agro-industrial pollution, and calling for the development of *eubiotic* governance and a stewarding of a microbial commons to support microbial health for the benefit of planetary flourishing.

A multispecies framework is a biocentric framework, but would it allow an ecocentric justice orientation? The former refers to entities understood as living in a certain, one might suggest limited, sense whereas the latter places intrinsic value on non-living things too, such as soil and waterways. In this logic of extensionism, an ethic of biocentrism implies recognition of the claims of more-than-human life. Yet insofar as such approaches continue to identify individuals as the only moral subjects, they have been seen as insufficient to the ethical transformation required to support the flourishing of environments. (Celermajer et al. 2021, 122; Boetzkes and Robert 2000). To address this specific lacuna, delegates to the First National People of Color Environmental Leadership Summit 1991, cited in Alston) in Washington DC, drafted and adopted 17 principles of Environmental Justice, for the growing grassroots movement for environmental justice (Alston 1991). As one of the organizers of the event reflected conveyed according to Alston (1991), for people of color, the environment is woven into an overall framework and understanding of social, racial, and economic justice. Environmental justice is an example of the power of principles in joining justice movements (cf. Donchin and Purdy 1999) and the accessibility and ethical sustainability of principles in a reconfigured framework (cf. Sudenkaarne 2021). The concept of environmental justice has come to address not only the classic issue of the social injustice of environmental impacts, but also the reality that “no system of social justice is possible without a functioning and sustainable environment” (Celermajer et al. 2021, 122). However, what we suggest is still one nudge further as environmental justice tends to focus on a functionalist approach to environments, albeit laudably offering a framework to interrogate environment-related social justice issues.

A multispecies approach to justice aims to tackle the fictitious idea of human beings as individual, isolated, unattached and unencumbered, and the correlative presumption that more-than-human nature is mere passive background (Celermajer et al. 2021, 120). It is an restorative effort to the violence generated by Western cosmologies that have divided human and non-human realms (Ishiyama and TallBear 2022; Author). It calls for adopting more relational ontologies for multispecies justice to recognize the multiplicity of different types of being, in their own terms and their involvement in thick relational webs, as rethinking the subject of justice moves attention from the fiction of individuals to the actual ecological array of relationships that sustain life (Celermajer et al. 2021, 120), and further, to new imaginaries of life also indifferent to human. Analytically, however, it can be argued that *multispecie*s is by definition in fact incompatible with rejecting human exceptionalism in a more thorough understanding, as the endeavor to compartmentalize life this way relies on human conceptualization of differentiating between species, to some in a similar hierarchy exercizsed in racism. So despite the myriad of laudable work done specifically with a multispecies justice orientation, we wish to further engage with One Health and more-than-human.

One Health is a stance building on the inseparability of the health of humans, non-human animals and the environment in a holistic understanding (Cañada et al. 2022, 1). Despite it gaining momentum over recent decades, for both AMR and CE, the potential of One Health often fails to deliver outside acknowledging different stakeholders and their interconnectedness to varying degrees, whilst human exceptionalism of the most privileged is uncontested (Cañada et al. 2022; Zinsstag et al. 2018).

For example, despite its One Health positioning, the rationale of the global AMR governance regime is one of protecting antibiotic efficacy for the purposes of human health, framed in terms of the rights of future [human] generations, with the oft-cited 2015 O’Neill Report (cf. Review of antimicrobial resistance 2016) warning that without action, tens of millions will be dying from currently treatable infections by 2050. More recently, the position of the environment in AMR governance has produced greater awareness of environmental ‘hotspots’—sites of greater bacterial and antimicrobial concentrations such as wastewaters and sewage, pharmaceutical and livestock production wastes—in driving resistance evolution has grown (Gillings 2017; Helliwell et al. 2021). Still, the motivation is geared towards preservation of antibiotic therapies for human health. The One Health mandate thus recognizes and acknowledges the sharing and circulation of microbiota between humans, non-human animals, and living environments (such as water, soils, food production), but it is human suffering that is understood to be ethically problematic. Non-human suffering as a result of AMR is given less moral worth and consideration.

Furthermore, One Health as it is currently performed is criticized for its top-down and unified version of scientific expertise, rather than demonstrating a serious commitment to the study of ontological multiplicity. This version of One Health rests on the foundations of global northern technoscience, with its idiosyncratic epistemic baggage and ontological commitments. It seeks a unified and singular response where there exists a plurality of climate, health and ecology configurations (Braverman 2022; Craddock and Hinchliffe 2015; Hinchliffe 2015). In this regard, One Health remains ambiguous in terms of moral theory—perhaps an emergent idea for ethical inquiries rather than a consistent ethical framework (van Herten et al. 2019). Our approach is eager to join forces with a One Health aim, albeit a reformulated one that fully appreciates the myriad of relationalities and perspectives that generate the diversity of life and health configurations globally.

To calibrate this momentum, we wish to build on the concept of more-than-human (e.g. Cañada and Venäläinen 2022). It is undoubtedly still anthropocentric in the sense that it has human as one of its denominators, but by the idea of *more than*, we not only wish to imply moral significance in the ecocentric and biocentric sense but also to include, in a network theory rather than confessional understanding, spectral realms such as ancestors, spirits, ghosts and deities that are crucial actors in several global sense-making systems (De La Cadena 2010). Making further way for the radical reorientation of human exceptionalism, by *more than* we wish to remain open to finding new ways of being with ethics that perhaps could be more attuned to planetary or even cosmological flourishing than current human-oriented approaches. To us, *more-than-human* interrogates anthropocentrism in moral significance and ethical analyses but does not wish to abandon or exceeds the human per se in a transhumanist understanding. We revisit this dynamic further in the last section on posthumanism, albeit unable to offer a full framework analysis here. It is crucial to note that to ground such a framework, justice and vulnerability are neighboring concepts: in our analysis of injustice, on many occasion it is precisely the detection and eradication of vulnerabilities that will pave the way to more just worlds. While a more thorough framework approach to vulnerability is offered elsewhere (Author in this special issue), suffice for now is that vulnerabilities can inform about injustices and vice versa; thus, with a more-than-human justice orientation and in a new ethical framework, analysis of vulnerabilities has the possibility to deliver *more than* privileged humans currently fathom or care to deliver.

We begin by discussing CE and AMR as super-wicked problems, to us not sufficiently attuned to a multispecies orientation of justice. To deliver the grounds for such an orientation we rather call more-than-human justice, we discuss Bangladesh’s export aquaculture (which demonstrates the need for a framework that is not human exceptionalist but can still compute social justice issues and structural injustice), and climate insecurity Ladakh, North-West India (where the existence of multiple ontologically distinct weathers offers fertile ground for envisioning a more-than-human social justice). We conclude by suggesting towards further consideration into a queer feminist posthuman framework for a more-than-human justice orientation. Firstly though, we discuss CE and AMR in the context of super-wickedness and the challenges for social justice.

## Justice and the super-wicked problems of antimicrobial resistance and climate emergency

Due to their inherently complex nature, global policy has approached both AMR and CE as wicked problems. The concept of a wicked problem has been applied to cumbersome challenges that cannot be solved using technical or policy and planning responses that were more or less standard in early social policy of liberal democracies (Rittel and Webber 1973). Instead, wicked problems are nebulous, awkwardly intersecting across different policy goals, and often lacking in a clear consensus in definition and solution for the problem (Littman et al. 2020; Rittel and Webber 1973). Littmann, et al. (2020) define both CE and AMR as “*super-wicked* problems”, global challenges that are even more complicated for policy for the following reasons: firstly, the urgency of the problems (time to solve them is running out); secondly, those seeking solutions are also part of the cause; thirdly, there is a weak or non-existent central authority; and finally, that policy responses “discount the future irrationally” (ibid, 426). For example, regarding the final point, the authors ask if it is rational in the long term to wait until the problem is critical before imposing coercive measures to limit antibiotic use. In policy terms, the problems is thus defined as one of individual behaviors requiring correction. This narrow rational framing remains anthropocentric and refuses the possibility of multiple climates, environments, and healths, and the multitude of ways that humans and non-humans relate to each other in diverse more-than-human sociocultural and sociomaterial ecologies that further enhance the super-wickedness of CE and AMR—an argument we pick up below. It is also risky in anthropocentric social justice terms, as imposing coercive measures is likely to negatively impact low-income countries and emerging economies and push already vulnerable populations further into poverty unless steps are taken to address the multiple lacks that antibiotics compensate for (Denyer Willis and Chandler 2019; Sariola et al. 2022).

Taking a dynamic systems approach, bioscientists have highlighted the critical question of whether reducing antibiotic use alone will tackle the problem of resistance, pointing to terrestrial and aquatic environments as reservoirs and even hotspots of microbial resistance evolution (Gillings 2017; Helliwell et al. 2021; Taylor et al. 2011). Here, the concern is that, despite AMR’s framing as a One Health issue in the Global Action Plan for AMR (WHO 2015), the environmental dimensions are neglected. The super-wickedness of AMR, and its entangled relationship with the CE becomes apparent when experts try to account for evolution and emergence of resistance in infinitely varied ecologies exhibiting different social and environmental characteristics, and with different evolutionary selection pressures. Microbial abundance and evolutionary pressures are exacerbated by warming temperatures, acidifying oceans, and the expelling of industrial and organic wastes into the environment, leading to hypernutrification and increasing microbial abundance. Furthermore, AMR evolution happens in the environment, with pathways of resistance traffic through which microbes travel driven by both anthropogenic and biotic activity. One Health has neither provoked a more ecologically enlightened engagement with the health of the biosphere that supports the life of the planet, nor an examination of moral responsibility beyond the anthropocentric need to protect antibiotic efficacy for human health, or to protect the health and wellbeing of animals and environments beyond their utility for human populations. An inherent feature of super-wicked problems is their globally scalar nature that defies any kind of standardized response. We require bioethical justice frameworks that can accommodate the local specificity, as well as taking seriously the kinds of multispecies rights that ensure vulnerable human, non-human populations, and their various intersections are acknowledged and ideally protected.

Crucially, Littmann et al. (2020, 436) note that instead of focusing on managerial, technical or medical matters, decision-making and policy-making around super-wicked problems are “inherently and inescapably ethical”. Further, justice is seen as a central norm or indeed principle guiding policy responses for both global challenges (Unesco 2017; Nuffield Council on Bioethics 2023). However, we insist that this decision = making needs *new* frameworks for justice, accompanied by moral theoretical consistency to make sure human forms of oppression are not reiterated in a more-than-human context. For example, the principlist approach suggested by Beuachamp and Childress (2019) defines justice as a group of norms to fairly distribute benefits, risks, and costs, linking justice to Rawlsian ideas of distributive justice (Tong 1996) accepted widely (Nuffield Council of Bioethics 2023) and considering justice as some kind of fairness. The dilemma is that in global comparison from a social scientific point of view, it is obvious that fairness is not the current state of affairs, not even as distribution of benefits, risks, and costs, let alone in more detailed ethical analysis. In fact, for both CE and AMR, their costs are the highest in conditions of extreme scarcity (McPherson 2021; Nuffield Council on Bioethics 2023), which constitutes the very opposite of fairness. Moreover, a distributive justice framework does not necessarily include multispecies, environmental let alone more-than-human agency, which we urge as a necessary condition for a framework of justice to actually achieve fairness. The notion that justice would be achieved in decision-making, policy-making and ethics-making without critical engagement with the limitations of currently dominant moral theories framing these processes is unfounded: a framework of common morality (cf. Beauchamp & Childress 2019) might be easily accessible but safeguarding against privilege and bias is not. This often leads to calls for justice “however one conceives of justice” (Littmann, Viens and Silva 2020, 441) which, when examining the track record of medical ethics from a decolonial, queer feminist crip vantage point, has indeed not been fair at all. So for us, any conception of justice simply will not do even “practically speaking” (cf. Littmann et al. 2020, 441) as it is unfounded to assume that the conception or framework for justice would not affect its application, particularly when not openly addressed, in biased ways favoring vantage points of those already privileged. If justice is defined and framed by those seeking common-morality pragmatics as “delicate balancing of benefits and burdens that will require difficult choices” (Littmann et al. 2020, 439), it is very likely those “difficult choices” continue to favor those with more social power.

Even though there does not have to be a moral theoretical consensus of justice to tackle injustices around super-wicked problems pragmatically, it can also be argued that the lack of ethical debate about justice is at the core of many injustice. We suggest a more-than-human orientation of justice could contribute to this debate. In the four-tier model of super-wicked problems suggested by Littmann et al. 2020, 426), a more-than-human justice orientation would, firstly, scale the understanding of time: the urgency to solve this is not only present urgency but cuts through more-than-human histories of flourish and threatens the future, glooming as more-than-human ecocide. Secondly, a more-than-human justice orientation interrogating human exceptionalism unequivocally shows how those (humans) seeking solutions are also part of the cause. Thirdly, a more-than-human justice orientation with a strong claim to framework development opens the question of weak or non-existent central authority to cover not only institutions and establishments but moreover, the ethical decision-making of those authorities. Finally, as discussed, a more-than-human justice orientation interrogates precisely the fairness of policy responses “that discount the future irrationally” (ibid.), but addressing how such analysis needs a new framework entirely. To further demonstrate why looking at AMR and CE together is crucial for justice, and further, to highlight the limitations of current justice frameworks, we will next discuss the Bangladesh’s export aquaculture to show how more-than-human actors already play an important role in environmental and climate politics.

## Wicked entanglements of climate and resistance: making a case for more-than-human justice

Climates are a multispecies endeavour, produced by and responsive to the presence, behaviours and activities of different forms of life that include microorganisms, plants, human and non-human animals. Microorganisms profoundly determine and affect geochemical processes in terrestrial, aquatic and atmospheric systems (Gillings and Paulsen 2014; Hird 2009; Kirchhelle 2023). Changes in the abundance of microorganisms are a consequence of human population expansion, alongside the expansion of plant, land animal and aquatic animal populations that have attended this growth, and which has significantly altered the abundance of microbial organisms involved in the cycling of carbon and nitrogen (Gillings and Paulsen 2014). This has accelerated exponentially in the past 100 years, with a quadrupling of the human population and the expansion of food production, energy production and resource extraction required to support them. Global warming and oceanic acidification have resulted from the intensification of industrial and agricultural pollutants and wastes, whilst the releasing of billions of tons of carbon into the atmosphere, has altered the size of microbial populations, along with their biochemical activity, and their community composition and dynamics.

Antimicrobial resistance occurs when bacteria equip themselves to thrive in threatening environmental situations by utilizing evolutionary mechanisms such as horizontal gene transfer (HGT). Microbial evolutionary selective pressures (the external force that causes a particular organism to positively adapt to environmental conditions) come from many places, including warming temperatures, changes in pH, exposure to pollutants and environmental degradation, and exposure to antibiotics. Anthropogenic activities increase the rate of HGT by creating the selective agents that drive what Gillings (2017) calls the bacterial SOS response. Humans, he argues, create hotspots of gene transfer from hospital, municipal, industrial and agricultural/livestock wastes, or by driving climatic and environmental perturbations (warming, acidification, increases in nutrient loading). These hotspots trigger the SOS response as bacterial communities respond to DNA damage by increasing the number of resistance genes available for acquisition, assembling them in plasmids or mobile genetic elements that traffic adaptive traits between species and strains. In this way, bacterial communities have produced a diversity of genes that were previously not available. This has profoundly changed the structure and gene content of microbial communities (Gillings and Paulsen 2014; Landecker 2016). Increasing disease emergence is also connected to climatic and environmental perturbations, whether that is microbial adaptations to anthropogenic activities resulting in pathogenic emergence (e.g. urbanization, deforestation, intensification of farming), or shifts and redistribution in abundance of pathogens resulting from natural disasters such as flooding, cyclones, drought and so forth — risking further exposure when antimicrobials are deployed to manage them.

To summarize, the climate is sensitive to microbial perturbations and changes in microbial abundance, which in the last 10,000 years have been driven by human practices such as agriculture and the domestication of a variety of plant and animal life (Gillings and Paulsen 2014). Likewise, microbes are sensitive to climatic disruptions and environment perturbations, to which they adapt through the exchange of genetic material. Humans and non-human animals are sensitive to these adaptations in microbial structure in terms of vulnerability to multidrug resistant infections and emerging superbugs. This would seem enough justification for a more fully realized operationalization of the One Health framework that takes fuller consideration of environments. When examined in the context of social and multispecies justice, however, the vulnerabilities for specific social groups are more pronounced, and the stakes for achieving a more-than-human operationalization of justice that refuses human exceptionalism, but without losing sight of development, gendered and racialized social vulnerabilities in human populations, are raised. In what follows, we shed further light on, albeit not exhausting, these aspects.

### The example of Bangladeshi export aquaculture

Bangladesh’s export aquaculture is a good example. Here we provide data from multidisciplinary collaboration undertaken in 2017 and 2018 between British and Bangladeshi social and bioscientists to assess antibiotic use in the sector and find ways to reduce it. An emerging economy of close to 175 million inhabitants (the majority of whom are vulnerable in social justice, economic and climatic terms), Bangladeshi aquaculture has become an important method of achieving food security, rural livelihood generation, and foreign currency generation. The majority of aquaculture is undertaken on what Belton and colleagues (2018) refer to as the “missing middle” of forms of varying sizes and characteristics that are neither properly traditional nor fully professionalized, but that contribute the bulk of seafood products for both domestic consumption and export. They are often the target of development interventions and stewardship programmes intended to improve awareness of AMR and the problems of antibiotic use (Thornber et al. 2020). Our study found that farmers experienced significant disease challenges, attributable to complex intersecting multispecies, climatic and socioeconomic vulnerabilities (Hinchliffe et al. 2018). Disease emergence and outbreaks are driven by intensifying climatic shocks, environmental degradation from pollutants (including pharmaceutical and antimicrobial pollution), increased nutrification of water, and general problems associated with managing low-tech, low-input ponds in situations of increasing urbanization, industrialization and market instability. Despite being a region vulnerable to high level of antimicrobial exposure (Hossain et al. 2017; McInnes et al. 2021), the research team discovered early on that antibiotics were rarely used to treat production problems.

Bangladesh’s shrimp and prawn sector began expanding in the 1970s following Independence in response to the increased global demand for high value crustaceans, and as a method for generating the foreign currency reserves required for the newly independent nation to develop. As of 2010, there were some 200,000 shrimp and prawn farms in Bangladesh’s southwest, covering an area of approximately 250,000 hectares (Belton et al. 2011, 25). The overwhelming majority are modified traditional ponds with some improvements in biosecurity, inputs and feed management. Farmers make a profitable but precarious living in a form of salvage accumulation (Tsing 2015), loosely tied into complex and informally organized supply chains, with production being converted into capitalist wealth at export stage, in processing factories controlled by powerful commercial actors (Hinchliffe et al. 2018). These risk averse lead firms amass the wealth, but do not involve themselves in production given the sector’s ongoing and increasing vulnerability to evolving lethal production diseases—resulting from a combination production intensification, its attendant proliferation of nutrients and organic loading, and increasing climatic instability and variability. Instead, risk remains in the hands of the farmers, who lack the financial resources and technical know-how to invest in improved biosecurity or pathogen control. The majority of farms, particularly in the coastal regions, operate open pond systems, where water exchange is tidal. Water is not treated before entering and leaving the ponds, and the conversion of land to aquaculture production has increased the nutrient and organic loading of receiving waters, already stressed from the discharge of wastes from towns and cities, terrestrial farming, hospitals, and energy and pharmaceutical industries (see also Butcher, forthcoming). Receiving waters are vulnerable to the kinds of organic wastes, toxic chemicals and heavy metals that create the environments for accelerated AMR evolution (Taylor et al. 2011; Watts et al. 2017). In short, the delta region has been transformed into a vast expanse of open farming systems that rely on coastal waters and river systems already receiving a high volume of untreated effluents from other industries, and facing severe water resilience issues (Mallik et al. 2018; Roy et al. 2018). Thus, whilst antibiotic use in Bangladeshi shrimp and prawn aquaculture is low and does not form a routine part of production practice, the nutrient wastes it produces combine with untreated water already contaminated with industrial and pharmaceutical pollutants, to create a potential resistance evolutionary hotspot, the effects of which are compounded by effects of the climate emergency. In AMR terms, it displays the characteristics of a resistance evolutionary hotspot, creating the conditions of bacteria, including pathogenic bacteria, to flourish and adapt. During interviews farmers described producing shrimp and prawn in an enduring state of pathogenicity, with pond environments that were increasingly hard to manage. They reported experiencing fluctuations in temperature or salinity, sudden drops in dissolved oxygen, or an overgrowth of algal blooms with putrid soils and foul-smelling black waters that they were unable to remedy (see also Butcher, forthcoming). They further reported experiencing unknown production diseases that they did not recognize and struggled to treat. The effects of increasing ocean temperatures and acidification created by the climate emergency (Ahmed et al. 2013) further stimulate the kinds of environmental perturbations that Gillings (2017) identified as precipitating the intensification of microbial self-defense mechanisms, creating the perfect conditions for resistance hotspots to emerge, the kinds of conditions that are used to justify top-down interventions aimed at changing individual behaviors, without engaging with the significant sociostructural challenges that determine farming conditions, and that limit the range of options available to farmers for improving their situations or modify their farming systems.

This example demonstrates how fundamental rights (in this case to food) are being undermined, and inequalities are being deepened by unstable environments created by microbial responses and CE, over which smallholder farmers have little agency to control. Moves to fully professionalized intensive farming systems (which come with their own sustainability issues and AMR risks, see Hinchliffe, Butcher and Rahman 2018), would only undermine these rights to food and livelihood securities, making smallholder farming inviable.

We thus encounter a seemingly intractable situation, requiring a conceptualization of justice that can provide urgent protection to prevent an environmental disaster, without excessively harming the already vulnerable human populations trying to eke out an existence in this market system of production and global distribution of food and wealth. Solutions will be difficult to find if sought within the system of resource exploitation and scientific technofix that produced them in the first place. It demonstrates instead a need for the kind of relational ethic proposed by for acknowledging not only human and non-human connectivity in AMR ecologies, but the situated condition and contingent nature of these ecologies that give rise to different hierarchies (or the perhaps relational priorities) in decisions over how to intervene. Kirchhelle (2023, 2) similarly recognizes the continuously evolving human-microbial relations in the midst of an antimicrobial era, which he recognizes as a “major signal of an unfolding planetary Antibiocene”. These shifts connect antimicrobial exposure and the climate emergency as urgent elements to be addressed in ecosocial or more-than-human justice frameworks.

This super-wickedness is compounded further when one takes into consideration the different subjective and affective ecological relations that human societies are embedded within, in which the ontologically diverse ways people relate to “nature” are distinct from the nature-culture positioning of Western political thought that underpins the global development governance. How to intervene in the climate and AMR emergencies when there is no consensus on the properties that constitute climate and microbes? Paradoxically, these are also spaces where, if sensitivity to local specificity can be taken seriously, the path towards a more-than-human orientation of social justice can be forged.

### The example of Ladakhi more-than human cosmopolitics

Anthropology, multispecies ethnography, and more-than-human geography offer conceptual approaches for diagramming the material-semiotics that evolve from the practical engagement of humans in more-than-human assemblages. For example, Jack et al. (2020) have asserted that scientists and policy makers overlook how are already indigenous values and worldviews are already defined by One Health principles. Anthropologist De La Cadena (2010) campaigns for the need to take seriously the public presence of non-human beings in the field of political activity. Drawing upon Stengers’ (2005, 995) conceptualization of cosmopolitics, where “cosmos refers to the unknown constituted by these multiple, divergent worlds and to the articulation of which they would eventually be capable”, she documents the involvement of more-than-human supernatural entities in Bolivian protest politics against resource extraction and environmental damage. De La Cadena (2010, 346) uses her observations to argue how their appearance may “inaugurate a different politics, plural not because they are enacted by bodies marked by gender, race, ethnicity, or sexuality demanding rights, or by environmentalists representing nature, but because they bring earth-beings to the political […]”. In his historical enquiry into the biosemiotics of Amerindian shamanism, Herrera (2018) similarly argues that, rather than being *discovered* by the scientific revolution, microbes were instead *observed and interpreted in novel ways* by the scientific revolution. Arguing that Amerindian shamanism has more in common with microbial ecology than the categories assigned to them by Western religious studies, he invites us to perceive the ‘cellular souls and microbial spirits’ inhabiting and participating in cosmologies of human and non-human animals, plants, soils, water sources, atmospheres and so forth. One of the Authors (Butcher 2021; 2017; 2015) has similarly examined the more-than-human ethics and relationalities that are instrumental in determining the fortunes of productive and reproductive activity in the Himalayan region of Ladakh, North-West India, relations that are becoming dangerously eroded in the context of neoliberal development, leading to the situations of dysbiosis and climatic disasters that characterize this current global epoch. We draw on some of insights produced by cosmopolitical and biosemiotic approaches here.

During fieldwork in Ladakh for her PhD, Andrea Butcher was searching for evidence of local deity participation in development activities, and had been struggling to find any. That changed on the night and morning of 5th and 6th August 2010, when the region experienced a series of cloudbursts, triggering destructive landslides and flash flooding that devastated local settlements (Butcher 2013; 2017). The causes were officially attributed to climate instability due to global warming. Locally, a further explanation existed alongside the scientific one: that both the tragedy and increasingly noticeable climate instability were due to retributive action of autochthonous supernatural inhabitants of the skies, land and water who —angered by an increase in environmental, ritual, and moral pollution resulting from unrestricted development; an introduction of novel livelihood practices; increasing material consumption and associated pollution; and a loss of respect for the Buddha’s teachings—sent the flood as punishment and warning.

Weather is of increasing concern for Ladakhis. A high altitude desert, its climate of bitterly cold, snowy winters and warm dry summers is giving way to warmer winters, wetter summers, and an increasing frequency in destructive storms such as the one of August 2010, creating anxieties for local populations and development organizations alike. A predominantly Tibetan Buddhist practicing society (albeit only marginally larger than its Muslim population), Ladakh is objectified as the quintessential model for sustainable development (as is the case with other Tibetan Buddhist societies). This objectification rests on a reinterpretation of Buddhism according to the epistemologies of Western normative scientific thought (and the ontological separation of nature and culture that underpin it) and secular liberal democracy. However, local engagement with “nature” signposts a qualitatively different relationship from the rationalized sustainable perspective. The critical reflection that followed in the disaster’s wake focused on the displeasure of more-than-human autochthonous guardians who share the domain: the mountain deities residing in the skies, the soil owners, and water spirits. Religious and productive life in Ladakh is dependent upon the protection and participation of these territorial guardians, who determine health, wealth, fertility and climate of the domain and its humans and nonhuman inhabitants. Mountain deities have jurisdiction over the weather and water, sending snow in the winter and sun in the spring to melt the snow and irrigate crops. Soil owners and water spirits are associated with fertility, abundance and nourishment. Frequently, mountain deities (and sometimes water spirits) are bound to Buddhism as guardians of the Dharma (religious law), and are thus also sensitive to a decline in observation of the Buddha’s teachings.

Post-flood, the author encountered an ethnographic situation whereby the developmental state of the Indian Union was supported in its ministrations by an older model of statecraft, in which development projects are accompanied by ritual offering to these more-than-human guardians and geomantic subjugation of negative forces. These included performances of Buddhist long-life ceremonies undertaken by prominent monastic leaders during tree planting festivals undertaken under the banner of “climate change remediation”, or the sponsorship of ritual technology such as the construction of devotional statues and stupas to prevent future disasters. Some NGO employees admitted to sponsoring small rituals. For example, ritual vases were placed at the foot of glaciers to appease the mountain guardians when diverting water to construct artificial glaciers (to supply freshwater for crop production in increasing situations of natural glacial retreat due to a warming climate). Sometimes they would sponsor religious exorcisms to remove demonic forces at sites of development constructions, such as schools. It may also be possible to reinterpret the symbolic underpinnings of these performances and relationships according to Herrera’s biosemiotics descriptions of environments where microbes reside (Butcher 2021). In this form of worldmaking, human personhood is conceived as chthonic (Mills 2003), embedded in ritual relations with a variety of divinities and numina—a situated and distributed chthonic agency, where absolute separation between humans and territorial divinities cannot be assumed.

What these ethnographic narrations drive home is that neither a distributive, undefined justice orientation nor a One Health justice orientation are sufficient or consistent enough to deliver justice, even as fairness, in this ontological multiplicity. As one of new framings in bioethics that we suggest is better suited to hone this multiplicity, we shall briefly discuss a queer feminist posthumanist framework.

## A queer feminist posthuman framework and the more-than-human justice orientation

Whilst arguably in the Ladakhi example, the more-than-human entities were delivering retributive justice, the ontological multiplicity running through these ethnographic accounts, and conceptual argument made by De La Cadena, correspond to an openness, flexibility, and predisposition to question what is normative. This repositions ways of knowing currently existing outside of the mainstream that are the essence of our queer, feminist bioethical framework. One Health is fundamentally reconceptualized here, with the environment foregrounded as a series of different entities to be engaged on their own terms, rather than rationalized as what is not, or other than, “human”, and significant only as far as they support human utility.

Building on this momentum, on the posthuman component of the framework, posthumanist contemplations overlap with classic questions in environmental philosophy, ecofeminism, and One Health (Wienhues 2017; Boetzkes and Robert 2000; Warren 2000). A common notion is that the quest for an inclusive health ethic has led to a critical examination of the conceptual constructions of categories like human/animal and living/non-living. Posthumanism can and, indeed, should inform the conversations around life, subjectivity, human nature, responsibility, and interspecies relations that underpin bioethical decision-making (Sands 2022, 1). These conversations state that how humans have conceived of health and nature must be revised, the denaturalized and decontextualized way we have understood ourselves as humans must be modified, and the prescriptions of ethics and prudence must be expanded, nuanced, and in some cases reconfigured (Boetzkes and Robert 2000, 150). To Boetzkes and Robert (ibid.), this reinforces what ecofeminists among others have long claimed: that progress in achieving “right relations” within ecosystems means scrutinizing inherited thought-forms, resisting the dichotomization of humans and nature, rejecting domination, and proposing intersectional norms of ethical behavior.

A queer feminist posthuman framework can call into question the apparently obvious coherence of human nature and aims to destabilize the basic premises of human exceptionalism^.^ with gender and sexual variance as a necessary condition in its moral theory. It also calls for serious reconfigurations of the collective human self: the metaphysical limits and ethical ramifications of “us” and the more-than-human. According to this line of critique, human ways of knowing and being in the world do not have privilege or priority over the myriad of nonhuman entities. As our ethnographic narratives discussed, significantly, it questions the identity of the human species as unified and self-present but thoroughly implicated in the phenomenology and ontology of more-than-human entities (Chiew 2014, 51 − 52; Barad 2007: 134 − 141), Yet crucially for an ethical framework and to enhance moral theoretical aims, its ontological conceptualization must go beyond the human condition with a justice orientation, akin to what Karen Barad (2007, 182; 391 − 396) calls entangled responsibility or accountability of intra-action as part of the fabric of the world, and what Donna Haraway (2016, 11; 13) calls tentacular response-ability and becoming-with. As Author (2023) has suggested, this framework urges asking what kinds of subjects can we imagine as morally significant. How are multispecies relations managed without prioritizing human ways of being? How are intersecting layers of vulnerabilities minimized between groups of people? Is species an oppressive category or in fact needed to ground subversive politics? How to move from human-oriented use of technology to solutions benefitting the entire global ecosystem? Crucially, a justice orientation more committed to nonhuman moral significance does not lessen human accountability. On the contrary, it means that accountability requires much more attentiveness to existing power asymmetries (Barad 2007, 219). An open, communal and collaborative process, building a queer feminist posthuman framework aims to make the case for posthuman moral responsibility, further enhancing the more-than-human justice orientation for AMR and CE policies.

A key position in the feminist orientation of the framework is the openness to lived experience informing theory, the ethics of everyday as embodied agents navigate it, as opposed to trickle-down approaches (Donchin and Purdy 1999). This includes allowance of what Haraway (2016) has called staying with the trouble: that there may well be conflicting interests and views present and that it is better to stay in dialogue, demanding answers, rather than to assume that a luke warm consensus would deliver any sustainable relief to an ethical dilemma that would not, if left unchecked, benefit the privileged position. This interrogation of normativity poignantly demonstrates how cis and heteronormativity operates hidden in moral theory and ethical deliberation and continues to warp our understanding of cosmological flourishing.

The Bangladeshi example highlighted, firstly, the interconnectedness of climate and AMR. Secondly, it demonstared the super-wickedness of CE and AMR in terms of caring for the environment and acknowledging the value of environments for their own sake in a One Health perspective, whilst simultaneously recognizing that Anthropos is not a unified entity, and that understanding vulnerabilities requires a nuanced analysis. The Ladakhi examples and cosmopolitics framings invite an openness and a questioning of normativity. To offer a parallel to Stengers’ notion of cosmopolitics, more-than-human supernatural entities to protest injustice (see also Govindrajan 2022), this framework can be further enriched by Gordon’s (2011; see also Sudenkaarne and Blell 2022) concept of haunting. To Gordon (2011, 2), haunting is how abusive systems of power make their impacts felt, especially when they are supposedly over and done with or when their oppressive nature is continuously denied. What is distinctive about haunting is that it is an animated state in which a repressed or unresolved social violence is making itself known, calling for transformation (ibid.) Haunting always registers the harm inflicted or the loss sustained by a social violence done in the past or being done in the present and is for this reason quite frightening. Haunting refers to a socio-political-psychological state when something else, or something different from before, feels like it must be done (ibid.). Ultimately, to us the Bangladeshi and Ladakhi case studies illustrate examples of "hauntings", urgent cries for things to be otherwise: offering an invitation to open up queer, feminist, posthuman perspectives in other words—and to other worlds.

## Conclusion

Considering AMR and CE as super-wicked problems has benefits but if accompanied with a non-defined or too narrowly defined justice orientation, it does little to offer ethical guidelines for tackling issues around AMR and CE for greater benefit, particularly in terms of cosmological flourishing. What we have suggested is a more-than-human justice orientation that, embedded in but not limited to a queer feminist posthuman framework for bioethics, seeks to protect and promote more just world-making.

## Data Availability

Not applicable.
